# Sensors for Environmental Monitoring and Food Safety

**DOI:** 10.3390/bios12060366

**Published:** 2022-05-26

**Authors:** Kevin C. Honeychurch, Martina Piano

**Affiliations:** 1 Department of Applied Sciences, University of the West of England, Frenchay Campus, Coldharbour Lane, Bristol BS16 1QY, UK; 2 Institute of Bio-Sensing Technology (IBST), University of the West of England, Bristol BS16 1QY, UK; martina.piano@uwe.ac.uk

## Introduction

The aim of this Special Issue of the journal *Biosensors*, “Sensors for Environmental Monitoring and Food Safety”, was to report on the developments and advances in sensors and biosensors to meet the needs of environmental and food analysis. Its objectives were to bring together a series of papers describing the advances and applications of both sensors and biosensors. The complexity of the environment offers a number of scientific challenges that are needed to be overcome to safeguard clean drinking water and food quality and to gain an understanding of the multifaceted world that surrounds us.

The present application of sensors for food and drink safety has been reviewed by Ferrari, Crapnell and Banks [1]. They have highlighted the need for robust, reliable, and affordable analytical techniques for the screening and monitoring of food and water quality. Their review investigates the promise that electrochemical biosensors offer for health and environmental monitoring and that these can provide an alternative solution to classical laboratory-based analytical techniques presently used. A large number of different electroanalytical sensor types have been produced for the detection of small molecules, proteins, and microorganisms utilizing different recognition systems, from direct electrochemical redox processes to biological recognition, antibodies, enzymes, and aptamers. They highlight that further work needs to be undertaken, with validation against standard laboratory-based techniques being needed.

A portable fluorescence resonance energy transfer (FRET)-based biosensor was reported by Lai et al. [2] for the determination of Pb in water. A 3D-printed frame was used to house a 405 nm laser diode as the excitation source to obtain fluorescence emission images that could be aligned with a smartphone. A limit of detection of 24 nM (4.74 ppb) was reported. To overcome possible interferences from elements such as Zn, samples were preincubated with tricine, a low-affinity Zn chelator. The method was validated with laboratory samples and water samples collected from six regions of Taiwan by inductively coupled plasma mass spectrometry. Two of the water samples were found to have Pb concentrations higher than that of the control used and were above the WHO-permitted level of 10 ppb for tap water.

Klimley [3] has reported on the application of sensors to investigate how scalloped hammerhead sharks can make their nightly migrations to feeding grounds as much as 20 km away. The possibility of the sharks utilizing differences in the intensity and type of light to navigate was investigated. Two sensors were developed to measure the irradiance intensity in the same spectral range and sensitivity of the shark’s cone and rods. The first sensor matched the photopic range using a photocell covered with a red-shifted gel filter; the second was matched to the scotopic range, in this case using a blue-shifted gel. The two sensors were attached to a shark that was tracked during its nightly foraging excursion to a distance of 20 km from its daytime abode, a seamount, in the Gulf of California. The depths at which the shark was swimming as well as the irradiance levels experienced were telemetered back to an onboard receiver and the possibility of the shark navigating using light was explored. However, it was found that the sharks rarely swam close enough to the surface for there to be sufficient light for navigation. Nevertheless, it was concluded that this paper illustrates the utility of adapting a sensor to investigate the systems that animals use and it illustrates the utility of using a multidisciplinary approach for sensor development.

Harmful algal blooms (HABs) are reportedly more frequent with tropical toxic species moving into higher latitudes as a result of climate change. Consequently, monitoring programs for detecting the presence of toxic algae before they bloom, are of paramount importance. Medlin et al. [4] have reviewed recent developments made in biosensor detection tools based on molecular barcodes. These were postulated as an alternative to the commonly employed microscopic counting-based techniques. Their electrochemical detection system was reportedly an improvement over conventional sandwich hybridization protocols. The application of magnetic microbeads and amperometric detection at screen-printed carbon electrodes to detect the target RNA of algae species required as few as ten cells/L for some species.

Similarly, the occurrences of cyanobacterial blooms are a threat to water quality and as a result, methods have been sorted to control them in the field. The Special Issue features a report by Li et al. [5] who have focused on the application of sonication for this. In their study, samples of cyanobacterial *Microcystis* were sonicated for differing times, and removal efficiency and changes in their morphology were investigated by transmission electron microscopy (TEM), polarized light scattering and spectrographically. Differing sonication times were found to be consistent with the removal efficiency and TEM images. The optimal sonication times were identified.

Miglione, Napoletano and Cinti [6] have reviewed the recent applications of in-the-field analytical tools for the determination of cyanotoxins in food and environmental matrices. The review gives an overview of the application of nanomaterials, synthetic receptors and microfabrication for electrochemical biosensors developed for the determination of the four cyanotoxins; microcystin-LR, anatoxin-a, saxitoxin and cylindrospermopsin. Their review highlights the advantages that electrochemical methods offer in terms of economics, ease of miniaturization, and overcoming elements such as color or turbidity which can be common interferences with spectrometric-based assays. The review concludes that there are still growing research areas and that major improvements can be achieved with a combination of emerging technologies such as; paper-based substrates, chemometrics/artificial intelligence, multi-recognition elements and the use of smart-nanomaterials.

A novel paper-based electrochemical aptasensor employing a tungsten disulfide (WS_2_)/aptamer hybrid has been reported [7] for the determination of the well-known foodborne pathogen *Listeria monocytogenes*. The sensor was reported to show a number of advantages, being simple, cost-effective, reliable, and disposable. The morphology of the sensor was characterized using a number of analytical techniques to investigate the optical, elemental composition, and phase properties of the synthesized WS_2_. Nanostructures were characterized by field-emission scanning electron microscopy, Raman spectroscopy, photoluminescence, and X-ray diffraction. Electrochemical impedance spectroscopy was performed to investigate whether the aptamer had been immobilized and also to assess the performance of the *L. monocytogenes* sensor. A detection limit and a limit of quantification of the developed aptasensor were reported to be 10 and 4.5 CFU/mL, respectively, within an associated linear range of between 10^1^ and 10^8^ CFU/mL. The proposed sensor was reported to be selective towards *Listeria monocytogenes* in the presence of other bacterial species such as *Escherichia coli* and *Bacillus subtilis*. The aptasensor was evaluated using a number of real samples fortified with concentrations of 10^1^, 10^3^, and 10^5^ *Listeria monocytogenes*.

A two-step electrochemical immunosensor was developed for the analysis of the peanut allergen Ara h 1 in a 1 h assay [8]. Bare screen-printed carbon electrodes were utilized as the transducer. Monoclonal capture and detection antibodies were applied in a sandwich-type immunoassay. A short assay time was achieved by the previous combining of the target analyte with the detection antibody. Core/shell CdSe@ZnS quantum dots were utilizsed as an electroactive label for the detection of the immunological interaction by differential pulse anodic stripping voltammetry. The linear range was reported to be between 25 and 1000 ng·mL^−1^ with an associated limit of detection of 3.5 ng·mL^−1^, and a sensitivity of 23.0 nA·mL·ng^−1^·cm^−2^. The immunosensor was able to detect Ara h 1 in a spiked allergen-free product down to 0.05% (m/m) of peanut. Commercial organically produced cookies and cereal and protein bars were investigated to track and quantify Ara h 1 and the results were validated by ELISA.

This Special Issue has shown the possible solutions that different sensor technologies can offer. Sensors and biosensors represent an attractive, efficient technology offering the possibility of rapid and reliable in the field applications for both the understanding of the environment and for the assessment of food and water quality.

## References

[1] Ferrari A.G.-M., Crapnell R.D., Banks C.E. (2021). Electroanalytical Overview: Electrochemical Sensing Platforms for Food and Drink Safety. Biosensors.

[2] Lai W.-Q., Chang Y.-F., Chou F.-N., Yang D.-M. (2022). Portable FRET-Based Biosensor Device for On-Site Lead Detection. Biosensors.

[3] Klimley A.P. (2021). A Sensor Designed to Record Underwater Irradiance with Concern for a Shark’s Spectral Sensitivity. Biosensors.

[4] Medlin L.K., Gamella M., Mengs G., Serafín V., Campuzano S., Pingarrón J.M. (2020). Advances in the Detection of Toxic Algae Using Electrochemical Biosensors. Biosensors.

[5] Li J., Zou C., Liao R., Peng L., Wang H., Guo Z., Ma H. (2021). Characterization of Intracellular Structure Changes of Microcystis under Sonication Treatment by Polarized Light Scattering. Biosensors.

[6] Miglione A., Napoletano M., Cinti S. (2021). Electrochemical Biosensors for Tracing Cyanotoxins in Food and Environmental Matrices. Biosensors.

[7] Mishra A., Pilloton R., Jain S., Roy S., Khanuja M., Mathur A., Narang J. (2022). Paper-Based Electrodes Conjugated with Tungsten Disulfide Nanostructure and Aptamer for Impedimetric Detection of Listeria monocytogenes. Biosensors.

[8] Freitas M., Nouws H.P.A., Delerue-Matos C. (2021). Voltammetric Immunosensor to Track a Major Peanut Allergen (Ara h 1) in Food Products Employing Quantum Dot Labels. Biosensors.

